# Supplementary material for: The effects of genital myiasis on the diversity of the vaginal microbiota in female Bactrian camels
Source: BMC Vet Res. 2022 Mar 5;18:87. doi: 10.1186/s12917-022-03189-5 (PMC8897907; doi:10.1186/s12917-022-03189-5)
Supplement: Supplementary file 5 — Additional file 5. [file 12917_2022_3189_MOESM5_ESM.zip › MPL201709200_16s_yy/Treat1/B13_bdiv/NMDS_weighted.pdf]

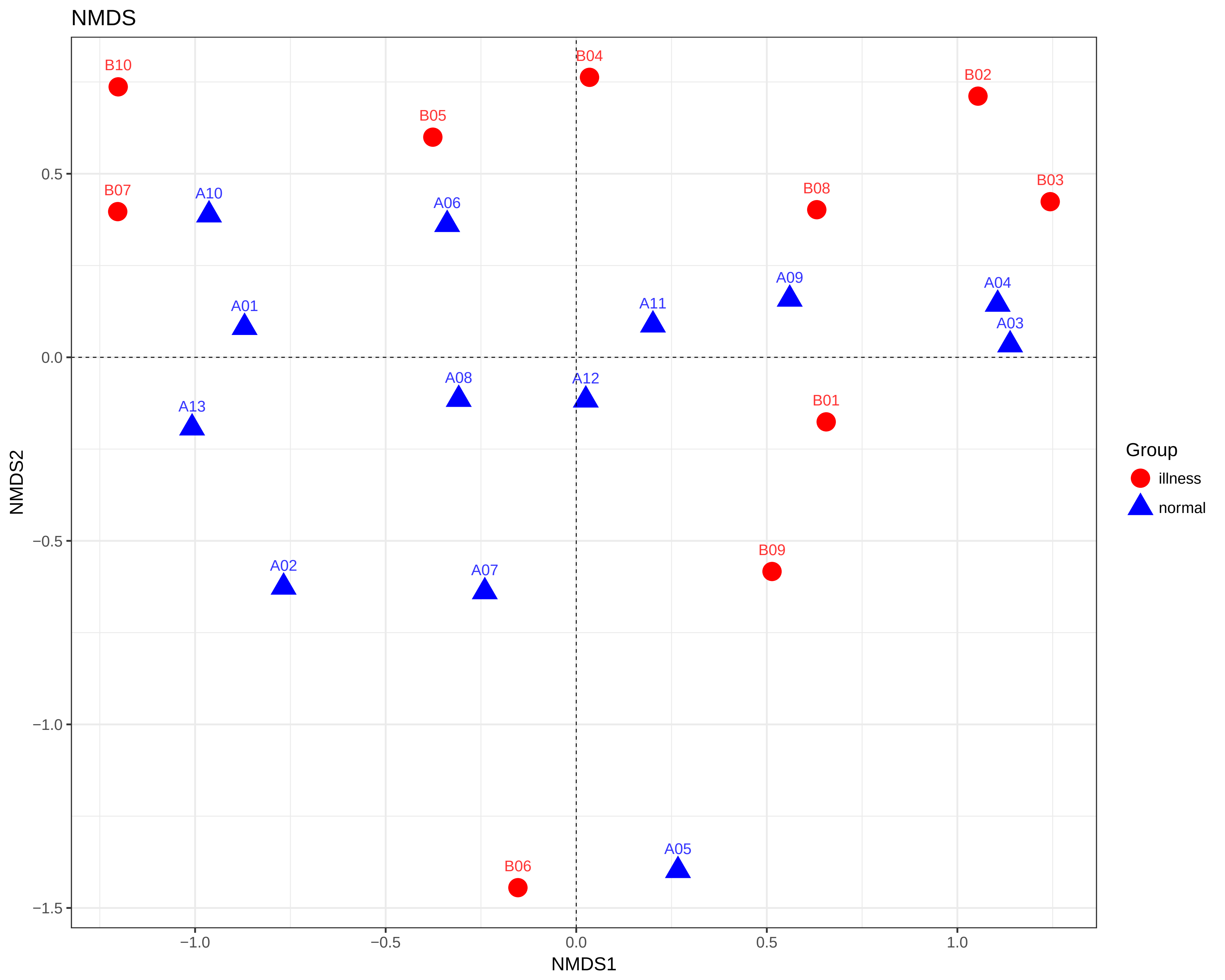

NMDS
NMDS1
NMDS2
−1.0
−0.5
0.0
0.5
1.0
−1.5
B10
B04
B02
B05
B07
A10
A06
B08
B03
A09
A04
A01
A11
A03
A08
A12
A13
B01
B09
A02
A07
A05
B06
Group
illness
normal
